# Time to Say Goodbye to Bronchiolitis, Viral Wheeze, Reactive Airways Disease, Wheeze Bronchitis and All That

**DOI:** 10.3389/fped.2020.00218

**Published:** 2020-05-05

**Authors:** Konstantinos Douros, Mark L. Everard

**Affiliations:** ^1^Third Department of Paediatrics, Attikon Hospital, University of Athens School of Medicine, Athens, Greece; ^2^Division of Paediatrics and Child Health, Perth Children's Hospital, University of Western Australia, Nedlands, WA, Australia

**Keywords:** viral, lower respiratory tract infection, neutrophil, infant, pre-school age children, wheeze, diagnostic feature

## Abstract

The diagnosis and management of infants and children with a significant viral lower respiratory tract illness remains the subject of much debate and little progress. Over the decades various terms for such illnesses have been in and fallen out of fashion or have evolved to mean different things to different clinicians. Terms such as “bronchiolitis,” “reactive airways disease,” “viral wheeze,” and many more are used to describe the same condition and the same term is frequently used to describe illnesses caused by completely different dominant pathologies. This lack of clarity is due, in large part, to a failure to understand the basic underlying inflammatory and associated processes and, in part, due to the lack of a simple test to identify a condition such as asthma. Moreover, there is a lack of insight into the fact that the same pathology can produce different clinical signs at different ages. The consequence is that terminology and fashions in treatment have tended to go around in circles. As was noted almost 60 years ago, amongst pre-school children with a viral LRTI and airways obstruction there are those with a “viral bronchitis” and those with asthma. In the former group, a neutrophil dominated inflammation response is responsible for the airways' obstruction whilst amongst asthmatics much of the obstruction is attributable to bronchoconstriction. The airways obstruction in the former group is predominantly caused by airways secretions and to some extent mucosal oedema (a “snotty lung”). These patients benefit from good supportive care including supplemental oxygen if required (though those with a pre-existing bacterial bronchitis will also benefit from antibiotics). For those with a viral exacerbation of asthma, characterized by bronchoconstriction combined with impaired b-agonist responsiveness, standard management of an exacerbation of asthma (including the use of steroids to re-establish bronchodilator responsiveness) represents optimal treatment. The difficulty is identifying which group a particular patient falls into. A proposed simplified approach to the nomenclature used to categorize virus associated LRTIs is presented based on an understanding of the underlying pathological processes and how these contribute to the physical signs.

“*When I use a word*,” Humpty Dumpty said in a rather scornful tone, “*it means just what I choose it to mean–neither more nor less*.”

*Through the Looking Glass Lewis Carroll* ***1871***

## Introduction

Respiratory viral infections are very common in the preschool years (1–4). It is now recognized that a large proportion of children acquiring a virus such as rhinovirus have no symptoms at all while (5–9) at the other end of the severity spectrum infants with viral lower respiratory infections may require mechanical ventilation and even die. Of these RSV, human metapneumovirus, adenovirus and influenza appear of most important (8, 9). Worldwide, respiratory viral infections remain a major cause of death. The respiratory syncytial virus alone is estimated to cause up to 200,000 deaths a year, predominantly amongst infants, with the vast majority of deaths occurring in “developing” countries (10) and while deaths from respiratory tract infections are falling significantly (11) in many areas they remain all too common. Fortunately, with good supportive care, viral respiratory tract infections do not kill large numbers of young patients in developed countries (12) though they remain amongst the most common cause of death in infancy in countries such as the U.K (13). However, viral respiratory tract infections are the major cause of ill health amongst infants and young children.

Given that most young children with a clinical “head cold” also cough and cough receptors are believed to be found exclusively in the lower airways this would suggest that in most cases of symptomatic infection viral replication extends from the nose and upper airways into the lower airways. A variety of factors determines whether the viral replication in the lower airways simply induces a mild bronchitis with coughing and some mucus production or has a more significant impact causing significant airways obstruction. Not uncommonly infants and young pre-school children with airflow obstruction may generate a “wheeze” in addition to increased respiratory rate and recession signifying significant airflow obstruction with a resultant increased “work of breathing” (expenditure of energy to move the chest wall). A wide range of terms have been used by clinicians to describe the “disease” a child with a viral lower respiratory tract infection is experiencing. Frequently these are purely incomplete descriptions such as “pre-school wheeze,” “happy wheezer,” or “reactive airways disease (RAD)” which specifically avoid trying to consider underlying pathology and indeed do not even acknowledge the role of the virus in acute episodes. A term such as RAD carries no real information given that the airways of any symptomatic child with a viral LRTI are manifesting a reaction to the presence of the virus whether this is predominantly a neutrophilic response or bronchoconstriction.

Others are descriptions of temporal patterns of symptoms that can only really be applied in retrospect such as “transient wheeze” (these temporal patterns are *definitely* not phenotypes even though this term is frequently misused in this context). Others such as bronchiolitis, bronchitis and larygotrachyobronchitis (croup) attempt to describe the region of the airway contributing most to the symptoms experienced.

The same term is often used for similar phenotypic appearances with completely different underlying pathologies such as the terms viral wheeze, wheezy bronchitis and RAD which have been used for both wheezing with a viral bronchitis and for a viral induced exacerbation of asthma in a pre-school child. Moreover, it is not uncommon for the same term to be used for completely different phenotypes such as acute bronchiolitis. In the North America and other countries following their lead, the term has apparently become reserved for a first *episode of wheezing with a viral infection in a child*<*2 years of age*. If there is a second similar episode, presumably with the same underlying pathology at play, they are then presumably deemed to have “RAD,” “pre-school asthma” “viral wheeze” or some other similar non-specific term. In the U.K., the term acute bronchiolitis has traditionally, but not uniformly, been reserved for very young infants with airflow obstruction and widespread *crackles* as was the case in early use of the term in North America (14).

As illustrated by Table 1 there are a huge range of terms used, often indiscriminately, by clinicians to describe respiratory illnesses induced by respiratory viral infections. Our failure to use language in a way that helps develop a clear approach to these conditions has contributed to our failure to significantly reduce levels of morbidity (modern medicine has had a huge impact on respiratory mortality in childhood but systemically fails to address morbidity in the same effective way). A conscious or unconscious recognition of this has, over the past 50 years, led to recurrent calls to revisit the way we discuss viral associated respiratory disease in infants and pre-school children (15–23). In North America there have been recent calls to abandon the use of “RAD” (19–21) but these have not been accompanied by a clear and logical approach to the use of diagnostic labels for respiratory illnesses cause by respiratory viruses in infancy and early childhood.

**Table 1:** Plethora of terms to describe acute lower respiratory tract infections experienced by infants and young children.

Viral lower respiratory tract infection
Acute viral bronchitis
Viral tracheitis
Group
Acute bronchiolitis
Viral pneumonia
Viral pneumonitis
Recurrent bronchiolitis
Wheezy bronchitis
Viral wheeze
Pre-school wheeze
Wheeze associated viral episode (WAVE)
Toddler wheeze
Happy wheezer
Recurrent wheeze
Pre-school asthma
Reactive airways disease (RAD)
Viral induced exacerbation of asthma
Transient wheeze
Transient asthma
Multi trigger wheeze
Exacerbation of persistent bacterial bronchitis

To be useful, medical terms need to convey information that is understandable to the person being addressed. This can only be the case if there is a common understanding of what a term means and its implications. As knowledge advances, terminology regarding clinical conditions should become more precise, carrying information that conveys information regarding pathophysiology, and information that clearly helps to institute optimal management. The intention of this review is to suggest a nomenclature based on the underlying pathophysiological processes contributing to the morbidity associated with acute viral infection and hence, most importantly, inform appropriate treatment decisions.

## Recognition of the Role of Viruses in Acute Lower Respiratory Tract Infections

By the beginning of the twentieth century it was widely recognized that a variety of bacteria appeared to play an important role in upper and lower airway infections. However, the concept of viruses was far from established despite the extraordinary insightful work and interpretation of the Dutch botanical microbiologist Beijerinck who published his work on the mosaic disease of the tobacco plant in 1896 (24). More than 30 years later the existence of viruses as a class of organism that were too small to see under the microscope and that required living host cells in order to replicate was still not universally accepted though a number of diseases such as rabies and influenza were thought to be attributable to viruses. Following the identification of influenza in pigs, human strains of influenza virus were finally identified in the early 1930's (25). A number of viruses and atypical organisms capable of causing respiratory illnesses were identified over the following 20 years. The big explosion in the identification of respiratory viruses occurred in the 1950 and 1960's with identification of respiratory syncytial virus, rhinovirus, adenovirus, parainfluenza, and others (1, 25–33). While a link was made between parainfluenza II and croup it was soon recognized that the majority of parainfluenza virus II infections were relatively mild respiratory illnesses while many other viruses were also able to cause croup. A similar story was soon apparent with RSV. Early studies noted that it was the commonest (but far from only virus) identified in infants (esp. amongst those aged 1–4 months) hospitalized with acute bronchiolitis but, it soon became apparent that it commonly caused little more than a “head cold” in the majority of infants and children. The list of viruses that are apparently capable of causing respiratory symptoms continues to grow.

## A Brief History of Nomenclature

Terminology for upper airways infections is relatively straight forward with the coryzal symptoms of a rhinitis, the inflamed tympanic associated with acute otitis media and the inflamed pharynx and/or tonsils of a pharyngitis or acute tonsillitis being generally easy to observe. Similarly, the hoarseness of laryngitis and the barking cough and stridor associated with croup in a young child should rarely cause diagnostic difficulties. However, this relative simplicity disappears when we consider the description of acute lower (intra-pulmonary) respiratory tract infection as shown in Table 1 with the greatest confusion occurring if wheeze is considered a feature.

The term acute bronchiolitis appears in the literature just before the middle of the C20th Holt and McIntosh observing in their textbook of 1933 that “*The symptoms are due chiefly to a bronchitis which extends into the smallest tubes. A fibrinous exudate, and in some case oedema, cause obstruction with great respiratory distress*,” (15). Even then there was considerable controversy as to whether it should be used at all and if so what features were specific for the condition or indeed whether this was just another name for bronchopneumonia (15). As more and more respiratory viruses were identified in the 1950 and 1960's it was recognized that most viruses could cause most clinical conditions leading to suggestions that “*clinicians and epidemiologists* ‘*throw away the book*' *in regard to previous descriptions of respiratory tract syndromes in children and start over as simply as possible*” (30). The authors suggested describing the illness on the basis of the location of the major observable pathology suggesting *tracheitis, bronchitis*, and *pneumonia*, or some combination of these were sufficient to describe the viral LRTI.

In 1973 Court noted that there was no generally accepted clinical classification of acute infective respiratory illness in children (17). He and his collaborators agreed a classification using “*traditional categories based on the part of the respiratory tract most severely affected*” which they modified following systematic application to children admitted *to hospital* based on a “*clinical descriptive or syndromal classification*.” The aim of the endeavor was stated as being to generate revised categories (which in practice differed relatively little from their starting position) “*in order to help improve communication between clinicians, provide a sharper tool for epidemiological studies and improve communication between students of these disorders*.” They described croup as a middle respiratory infection and had three categories of lower respiratory infections (a) acute bronchitis (mainly affecting children <6 years, cough being constant, wheezing, and breathlessness very frequent i.e., >50% of case); (b) acute bronchiolitis and (c) pneumonia. The same issue of the journal contained a number of other articles along the same lines applying criteria to patients seen in hospital and/or primary care. Curiously, in one of these studies (18) the definitions used by GPs and hospital staff varied significantly while many of the signs reported by GPs such as “*localized or generalized moist sounds*” did not appear in the definitions. “Bronchitis” in children admitted to hospital was frequently associated with “wheeze” but in primary care purulent secretions was far more common and “wheeze” not reported to be a common feature. All of the articles focused on acute illness associated with an apparent infection and specifically excluded asthmatic “attacks” precipitated by other causes. They specifically avoided mentioning infectious agents, while viral exacerbation of asthma were not mentioned.

By the late 1980's the concept that all wheezing children had “asthma” was adopted by many based largely on a study in primary school aged children which suggested that those with asthma and wheezy bronchitis were indistinguishable (34) and other studies emphasizing the under diagnosis of asthma in primary school age children who were missing out on effective therapy (35). These observations were somewhat uncritically extrapolated to infants and pre-school children. This change in the prevailing “fashion” in diagnostic labeling and the associated diagnostic transfer resulted in a very large increase in admissions to hospital for pre-school “asthma” which was mirrored by a fall of equal magnitude in other diagnostic labels for pre-school LRTIs (36).

However, by the mid 1990's, the recognition that the majority of pre-school children who have a tendency to wheeze with respiratory viruses tend to outgrow this tendency led to an explosion of terms to describe acute lower respiratory tract infections associated with airflow obstruction (often manifest by wheezing) (37). This resulted in a boom in papers claiming to describe different “phenotypes” of preschool wheeze which, as noted above, were just describing temporal patterns of symptoms (37–39). In large part this was driven by a sometimes explicit, sometime subconscious recognition that a proportion of children did develop asthma in the early years of life but that they were outnumbered by those who wheezed but did not have asthma and that formulating clear guidance on management was/is a challenge.

In the following section we would suggest that a classification that recognizes the factors that might contribute to airflow limitation in young children might prove to be far more productive in helping promote effective dialogue and approaches to treatment. However, in order to reach this point it is important to discuss factors that influence the phenotypic appearance of an acute illness and those that affect severity.

## The “Snotty Lung”–why it Causes so Much Confusion

A key observation that is central to the understanding of viral induced respiratory disease is that the inflammatory response in both the upper and lower airways are characterized and dominated by an airways neutrophilia.

**Figure 1:**
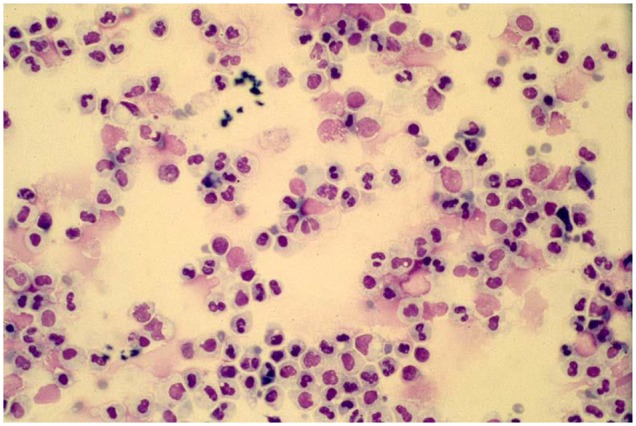
A typical cytospin preparation of a bronchoalveolar specimen obtained from an infant with respiratory syncytial virus bronchiolitis illustrating the intense neutrophilic response typical of symptomatic respiratory viral infections.

While in many clinicians minds viral infections are associated with a lymphocytic response a key to understanding the various manifestations of acute respiratory illnesses caused by respiratory viruses is to recognize that these infections are characterized by an intense airways neutrophilia and that most manifestations of the infection are caused by this host response. Some 50 years ago it was shown that the increase in peripheral blood white cell counts during experimental respiratory viral infections in adult volunteers was attributable to an increase in circulating neutrophil numbers (40). The importance of the intense airways neutrophilia in generating symptoms was recognized in a study of infants hospitalized with RSV bronchiolitis in the 1990's (Figure 1) (41–43). This neutrophilia appears to be driven by epithelial cells releasing cytokines such as IL-8 and neutrophil numbers appear to peak at or very close to the peak of symptoms (42–44). Neutrophils release a number of potent inflammatory agents such as human neutrophil elastase, myeloperoxidase, and metalloproteins (43) that drive the release of mucus, transudation of inflammatory fluid, mucosal oedema, coughing, and sneezing. The common feature shared by the “respiratory viruses” appears to be their ability to generate a neutrophilic response that helps promote the dissemination of the virus through airborne droplets (41–50). The viruses use a variety of approaches to deal with host responses such as the major antigenic shifts manifest by influenza, the existence of well over a 100 of serotypes of rhinovirus (51) and the ability of RSV to prevent effective herd immunity by preventing the development of long-term effective immunity (52, 53). However, they share a common approach to enable their dissemination, that is their ability to induce a neutrophilic response that drives the host to involuntarily generate bioaerosols. While neutrophils clearly drive symptoms, it is still unclear whether they have any role in limiting viral replication.

The importance of neutrophils in the generation of symptoms was clearly demonstrated in adult volunteer rhinovirus experiments in which only those subjects who generated a neutrophilic response developed symptoms even though viral replication could be demonstrated in the majority of the asymptomatic subjects (54–59). While these studies were undertaken in adults, they pointed to the fact that not all virus infections generate symptoms. As noted above, this insight has been confirmed in many recent studies in which a significant proportion of infants and young children are found to have evidence of acquiring a virus but do not exhibit symptoms (5–8). This is particularly common with rhinovirus but very uncommon with RSV suggesting that there are both host and viral factors at play.

While there is clear evidence that a neutrophilic response may be of benefit to the virus assisting in spread to other subjects, there is no convincing evidence that the neutrophils contribute significantly to clearance of the virus. Amongst asymptomatic subjects the virus is cleared with little or no significant neutrophilic response.

The severity of any viral “LRTI” is likely to be influenced by many factors which can be considered to be:-

1. those influencing the magnitude of the neutrophil dominated inflammatory response
2. those modifying the impact of the inflammatory response on function of the conducting airways and distal gas exchange region of the lung.

1. Factors Likely to Influence the Intensity of the Neutrophilic Response and Hence Symptoms
  a) Type of Virus
    Identification of the presence of a virus in the upper airways of a child with an acute respiratory illness generally leads to an assumption that the virus is causative. Recent studies have shown that for some viruses such as RSV this appears a reasonable supposition given that it is rarely identified in asymptomatic subjects while for others such as rhinovirus the rate of isolation in symptomatic children is barely greater than in asymptomatic children (5, 7). This does not necessarily imply that the latter is not an important player in a young child with an acute respiratory illness but may simply reflect the fact that other factors beyond just acquiring the virus need to be in play for the illness to be symptomatic. For RSV the lack of long-term effective immunity (60) may contribute to the development of symptoms with each infection while for rhinovirus factors as differences in the microbiota of the airway maybe important as noted below.
    Observational studies have suggested that LRTIs associated with RSV or hMPnV for instance do cause more severe disease in infants than for example hRv (61–65). However, this again does not prove that these viruses are innately more pathogenic as severity of any illness will be influenced by other factors such as maternal acquired immunity in the early months.
  b)Viral Load
    The role of viral load in disease severity is far from clear with studies suggesting variously that viral load has no effect on disease severity, predicts disease severity for some viruses (66–68) and not others (69). However, others have reported that a high viral load at presentation is associated with more rapid recovery (70, 71). The viral load in patients with disease will of course be influenced by a variety of factors including the initial infecting dose, innate defensive responses and more specific memory antiviral responses with many suggesting that the importance of viral load varies with the virus.
    It seems probable, but unproved, that the initial infecting dose may influence the severity of a clinical illness. Animal models of infection appear to support this suggestion while the natural experiment undertaken by practicing clinicians would also seem to support this. A common experience amongst pediatricians is that early in their career they experience multiple respiratory (and other) infections. They then appear to be “immune” to respiratory infections experiencing few if any until they have their own children a who kindly share their viruses with the rest of the family. The higher infecting dose associated with the intimate contact of cuddles and so forth once again result in symptomatic infections.
    The incubation period between inoculation and symptoms developing is generally in the realm of 2–5 days. During this period the rate of replication will presumably be influenced by the initial viral load at acquisition, subsequent top ups from siblings or day care attendance and host responses such that the “viral load” when measured during the acute illness need not reflect the initial “infecting dose.”
    While a number of studies have suggested that a higher viral load is seen in those most severely affected the overlap with levels in mildly affected patients is very large. Others have proposed that a high initial load at presentation is associated with milder and shorter illness through initiating a more robust response but given the very short timelines this is questionable.
  c) Co-infections With Other Viruses
    At present the evidence suggests that co-infections with two or more respiratory viruses probably do not generate more severe infections and associated increase inflammatory responses with some studies suggesting that co-infections generate lower levels of inflammation (72–77). There are a number of studies that do suggest a more severe illness with two or more studies though the reason is unclear. This may be related to specific viruses but without concurrent community data to act as a denominator information obtained in secondary and tertiary care centers is likely to be prone to systematic inaccuracies.
  d)Viral–bacterial Interactions
    In contrast to the lack of evidence supporting the suggestion that the presence of multiple viruses would produce a more severe disease there is a growing body of evidence that clearly implicates viral-bacterial interactions as a key determinant of disease severity both in the upper and lower airways. In a study undertaken amongst pre-school children attending a day-care center the severity of the official SNOT score, denoting the degree of nasal discharge, during an apparent viral infection was not influenced by the type of virus but rather the presence and density of H Inf and Strep Pneumoniae cultured from the nose (78). Subsequent studies including studies of the resident microbiome have provided supporting evidence for the importance of both these organisms and others such as Moraxella in the generation of symptoms during an acute “viral” respiratory illness (79– 84). As we learn more about the interactions of bacteria, viruses, and other organisms as they seek to obtain an advantage in a given niche the more the discrete distinction between viral and bacterial infections break down.
    These organisms are present in the “healthy” microbiome of children and given the vagaries of traditional microbiology the “commensal” organisms cultured from the upper airways of infants and children may simply be part of the microbial population of that child.
    To date vaccinations for “respiratory viruses,” other than influenza have been disappointing for a number of reasons. The only vaccine that has apparently significantly reduced the rate of hospitalization for “viral lower respiratory tract infections” including those caused by RSV has been conjugate pneumococcal vaccine as noted from a large study in South Africa (85). This again suggest that bacteria may influence the severity of “viral” LRTIs.
    Intercurrent viral infections appear to be a very important trigger of acute exacerbations amongst those with a persistent endobronchial infection such as young children with “PBB” (with or without radiologically proven bronchiectasis) (86–89), including those with conditions such as cystic fibrosis (90) and adults with COPD (91) again emphasizing the interaction of viruses and bacteria within the airways. As with asthma, a viral infection will lead to a significant increase in symptoms which is likely to result from the biofilms changing their behavior in response to the viral infection and releasing increased number of planktonic organisms. Strong support for this suggestion was provided in an *in vitro* study in which a biofim of PsA had been established on cultured differentiated CF epithelial cells (92). Addition of rhinovirus resulted in release, dispersal and migration of planktonic bacteria which is likely to enhance inflammation. From the bacteria's point of view, the increased mucous and airways fluid production together with coughing provides a very good opportunity to not only increase the colonization of the airway but, provide a means of dispersal that may permit colonization of another host. A key mechanism for both enhanced symptoms in an otherwise healthy child and in the child with and endobronchial infection is likely to be through increased free bacterial load adding to the neutrophilic response. As we discover more about polymicrobial infections many other interactions are and will be identified (93–104).
    Our understanding of the role that the respiratory microbiome (which varies across the varying niches from nose to distal airway) and that of the gut in promoting health and disease within the airways is still at a relatively early stage (105–107). What seems clear is that our relatively simplistic views of a single organism causing a given acute illness, which is largely based on Kock's postulates formulated more than 120 years ago (108), grossly oversimplifies the complex interactions of individual microorganisms with other members of the microbiome and the host. These “postulates” served us well in the early days of microbiology helping to clarify the role of virulent pathogens that often caused life threatening disease such as pneumococcus, meningococcus, diphtheria, TB, and so on but they break down in the less severe and often chronic illnesses including those involving biofilms and interactions between two or more micoorganisms (109, 110).
  e) Host Immunity
    Though seldom considered there is likely to be innate natural variation in the magnitude and effectiveness of neutrophilic responses between individuals which may contribute to observed variations in the likelihood of a young child developing symptoms such as a snotty nose. For instance, our unpublished *in vitro data* showed outliers in the release of inflammatory mediators when the neutrophils from healthy volunteers were exposed to a range of stimuli. In the absence of any firm data we would anticipate this is *probably* a relatively small factor in determining disease severity.
    Infants benefit from passively acquired maternal antibodies which help to protect against severe disease, particularly in the most vulnerable early months of infancy (111–113). The levels of antibodies required to prevent significant LRTI infection is an order of magnitude lower than that required to protect the upper airways as exemplified by the commercially available anti-RSV monoclonal preparation which significantly reduces clinically significant lower LRTIs in at risk infants but has little effect on upper airways infection (114, 115). Low levels of RSV antibodies in cord blood is common and represents a risk factor for severe disease presumably due to the poor memory responses in mothers resulting in many/most infants being very vulnerable in the early months of life. For many other viruses the passively acquired antibodies provide significant protection against severe LRTIs in the very vulnerable early months of life.
    Maternal antibody levels fall through the early months of life as the child's own immune system starts to take the strain producing specific antibodies with each exposure to a novel virus. Increasingly the exposure is to previously encountered viruses and/or related strains and the numbers of significant LRTIs (in the absence of comorbidity such as asthma or PBB) fall through the pre-school years.
    The role of “innate immunity” (116, 117) (other than that of neutrophils) in susceptibility to viruses and subsequent risk of a superadded bacterial bronchitis being established is unclear. While clearly not as an important risk factor for significant pulmonary disease as antibody deficiencies, defects in the innate immune system may play an unrecognized role in determining why some infants and young children go on to develop a persistent bacterial bronchitis while the majority of those with a viral LRTI recover without significant sequalae. Even if this is the case it would not influence current therapy.

2 Factors That Influence the Impact of a Given Neutrophilic Response
  a) Post Natal Age and Physiological Development Influencing Phenotype of Lower Airways Disease
    Infancy and early childhood is a period of great change with rapid changes in cognitive development, physical size, motor function, and so on. A 4-years-old pre-school child is very different to his 2-months-old self. These changes affect virtually every aspect of the individual including the structure of the lung and airways (118–124). The most obvious change is an increase in caliber of the airways which essentially completed their development in terms of branching before birth and a dramatic increase in surface area available for gas exchange resulting from increased numbers of alveoli and associated ducts. Another development that distinguishes the two are the numbers of pores of Kohn which are virtually absent in early infancy and early childhood appearing in significant numbers by the late pre-school age group. These differences influence the severity of symptoms experienced for a given level of inflammatory response. The absence of pores of Kohn accounts in large part for the common finding of partial or complete collapse/consolidation amongst infants with “acute bronchiolitis” affecting the right upper and/or right middle lobes in particular (the reason chest x-rays are inappropriate in most infants a “typical bronchiolitis” as these appearance frequently induce less experienced clinicians to prescribe antibiotics).
    The physical dimensions of the infant's airways make them much more susceptible to developing airflow limitation with resistance to flow being inversely proportional to the cube of the radius (1/r^4^). Similarly, the lack of pores of Kohn makes gas trapping and/or atelectasis acutely much more likely. Hence the same inflammatory response is likely to have a much greater impact on the intrapulmonary resistance and consequently the work of breathing in an infant than will be the case for a 6-years-old.
    As well as affecting the severity of the clinical illness experienced these same anatomical changes also result in very different auscultatory and auditory signs despite the same underling pattern of inflammation. This will be discussed below.
  b) Airways Abnormalities
    The story of repeated “bronchiolitis” and “recurrent troublesome wheeze” in infancy and early childhood is highly suggestive of an airways abnormality, most commonly a variant of tracheobronchomalacia (125– 127). Once an infant has been admitted four or five times in the early months of life with “bronchiolitis” or similar diagnostic label, the probability of an underlying airways is highly probable. Extra-thoracic issues such as laryngomalacia and sub-glottic stenosis more typically have a stridulously component, the upper trachea being extra thoracic.
  c) Co-existing Disease
    - Chronic Lung Disease and Congenital Heart Disease
      It is widely recognized that infants with CLD of prematurity and other underlying forms of CLD such as the various forms of interstitial lung diseases cope less well with viral LRTIs than previously healthy children with a disproportionately high rate of admissions to hospital (128–133).
    - Persistent Bacterial Bronchitis
      This condition, which has existed for centuries, most commonly develops in infancy and early childhood (86–89). The initiating event commonly appears to be a significant viral LRTI with the cough failing to resolve. The typical chronic inflammatory response to the persistence of bacteria such as Strep Pn, NTHi, and Moraxella is a sustained airways neutrophilia. While these appear to be part of the normal microbiome, disease appears to result when the diversity of the microbiome narrows and certain “pathogens” form a disproportionate component of the residual microbiome presumably through mechanisms such as the formation of biofilms and inhabiting intracellular niches. With intercurrent viral illnesses there is a significant flare up of symptoms presumably due to the interactions of bacteria, viruses and host responses as noted above. Typically, the wet cough becomes significantly worse while in younger subjects they may also develop significant airways obstruction which may result in wheeze as well as “ruttles” and indeed an oxygen requirement. Untreated, the symptoms tend to regress to the mean background level of coughing but will generally not resolve completely without aggressive antibiotic therapy.
    - Asthma
      Asthma appears to be the acquired loss of homeostatic control of airways smooth muscle (ASM) in post-natal life (134). The defining feature of asthma is loss of the effective homeostatic that develops prior to birth that maintains a stable airway and prevents excessive ASM shortening that can result in significant airflow obstruction. As such the diagnostic label denotes a discrete disease and should not be used as a catch-all for a child with wheeze–a symptom that can have many causes. Inflammation (atopic or otherwise) is neither sufficient or necessary to develop asthma though it may often accompany it. This is analogous to cystic fibrosis lung disease which is caused by a defect in the homeostatic mechanisms controlling airways surface hydration. Infection and inflammation are a consequence not the cause of the disease.
      Loss of ASM homeostasis can occur at any age. Epidemiological studies suggest that the majority of adults with asthma develop the condition in adult life. Quite how homeostatic control is lost is unclear but rarely appears to occur before the end of the 1st year of life. Studies have clearly shown that infants are born with functional ASM which equals or exceeds the relative mass in adult airways (134). Given that the ASM has been active through most of infancy playing a critical role in airways development and branching this is not surprising (135, 136). Studies undertaken more than 20 years ago showed (again unsurprisingly) the infant airway responds to agents such as histamine and methacholine and that short acting b-agonists can protect against this induced bronchoconstriction (137–142). They also indicated that b-agonists could lead to increased flow limitation rather than produce significant bronchodilation (141, 142). It is also important to recognize these agents were delivered as aerosols and the resultant physiological effect indicated that inhaled aerosolised drug does penetrate to the lower airways very effectively. *In vitro* and deposition studies suggest that the *dose per kilogram* reaching the lungs when using a nebuliser or pMDI with spacer and facemask (in a complaint infant) is likely to be at least as high in infants as in adults (143, 144). These studies should have put to bed urban myths such as b-agonists do not work in infancy because “*they do not have sufficient airways smooth muscle*” or “*they do not have effective b-receptors*” *or* “*because insufficient aerosol is inhaled because of their small tidal volume*.” Hence the observations that symptomatic infants rarely respond significantly to b-agonists and the lung function studies failing to demonstrate improvement in symptomatic children (145–147) probably reflects the fact that asthma is rare in infancy and that symptomatic infants are unlikely to respond to “asthma medication.”
      However, the caveat is that during an acute viral induced exacerbation of asthma b-receptor responsive is lost to a variable extent. Hence a viral induced LRTI in a non-asthmatic pre-school child generating sufficient airways secretions and oedema to generate airflow obstruction can appear very similar to an asthmatic with a viral exacerbation who is responding poorly to their b-agonist. An exacerbation is fundamentally different from poor control. An untreated or poorly adherent asthmatic generally responds well to a b-agonist when they have exercise induced or nocturnal symptoms but during an exacerbation the response to a b-agonist can be very poor (135, 148) and require systemic steroids to restore b-agonist responsiveness and relieve the ASM spasm. If there were a good response, asthmatics would not be admitted to hospital nor require corticosteroids. The mechanism that leads to loss of good b-agonist responsiveness is unclear but may be attributable to the impact of viral RNA (135, 149).
      Since the exacerbations are normally precipitated by viral infections it is not surprising that a neutrophilic response is generally observed. Neutrophilic inflammation does not respond to corticosteroid therapy explaining the lack of benefit when used to treat non-asthmatic symptomatic LRTIs. The re-establishment of bronchodilator responsiveness as a result of corticosteroid therapy in exacerbation of asthma is quite possibly the result of a direct effect on ASM rather than due to their “anti-inflammatory” effects.
      Given that asthmatics characteristically do not respond dramatically to bronchodilators during acute exacerbation and those with a viral LRTI have an intense neutrophilic response causing airflow limitation also unresponsive to bronchodilators the response to b-agonist therapy during an acute episode is common to both. However, the response to corticosteroid therapy is likely to be different with asthmatics likely to benefit. The challenge for clinicians therefore is to identify those who may benefit while not over treating those who will not.

## The Impact of Age on Symptoms and Signs

Pediatricians should be only too aware of the potential impact of age on manifestation of a disease. A good example might be coeliac disease. A common underlying immunopathology can result in superficially very different clinical pictures at different ages. An infant may present with “classical” failure to thrive, miserable, potbellied with gross diarrhea. A late pre-school failure my present with growth failure, their height falling away from the percentiles and be suspected of having growth hormone deficiency. In the late teenage years, the same pathology may present with lethargy, general malaise and anemia with symptoms being dismissed as “typical teenager,” “depression,” “too much alcohol ingestion,” or multitude of other mis-diagnoses. The individual only gets their life back when the correct diagnosis is made.

The same applies to viral LRTIs in the early years of life. The same inflammatory response dominated by neutrophilia leads to coughing and the production of airways secretions and some mucosal oedema. The airways secretions in particular may significantly increase the resistance to airflow within the conducting airways which may range from mild to severe. This change maybe manifest by signs such as tachypnoea, subcostal, and suprasternal recession, grunting, and hyperinflation with the presence, and magnitude of each symptom depending on the severity of obstruction. Grunting is much less common in older pre-school children than amongst infants, but all of these signs may be manifest by a pre-school child of any age with a significant LRTI.

While the above signs cause little confusion with most clinicians being able to interpret them as being indicative of increased work of breathing it is interpretation of the adventitial breath sounds that seems to lie at the heart of the confusion surrounding thinking about viral LRTIs in infants and pre-school children with and without “wheeze.” Confusion regarding the interpretation of breath sounds is not novel being evident in the earliest translations of Lannec's great work of 1819 (150). In order to get to the bottom of this confusion it is necessary to understand how the different breath sounds are generated and their significance.

## Generation of Adventitial Respiratory Sounds

Adventitial sounds can be inspiratory, expiratory or both (150–156). Extra-thoracic obstruction such as laryngomalacia and subglottic stenosis is generally worse on inspiration as the negative intrathoracic pressure draws in air and tissues in the extra-thoracic airways while dilating the intrathoracic airways. Hence inflammation predominantly affecting the “mid-airways” such as that associated with the viral respiratory tract infections exacerbating laryngomalacia or causing “croup” (in which the maximal narrowing is typically in the immediate subglottic region) predominantly generate an *inspiratory* stridor.

A **wheeze** is a generally expiratory musical sound generated by *flow limitation* affecting the lower airways, not “*turbulent airflow through narrowed tubes*” (151, 154, 157). As most medical students are taught “*all that* ‘*wheezes*' *is not asthma*” with flow limitation due a wide range of causes potentially causing wheeze. Its origins are attributed to the “father” of bronchoscopy Chevalier Jackson, used when highlighting the fact that a wheeze may be due to an inhaled foreign body. The mechanism leading to the generation of a wheeze appears to be vibration of the walls of the larger central airways as a means of dissipating excess energy from the system. Generally, exhalation is a passive process dependent predominantly on elastic recoil of the lung tissues, the potential energy added to the system during inhalation being translated into movement of air out of the lungs as it is released. The efficiency of the system is demonstrated by the fact that sounds associated with exhalation are generally very quiet and limited to the early phase of exhalation. In the face of flow limitation, the passive recoil provides more energy than can be accommodated by movement of air and the excess is dissipated by vibration of the central airways which manifests as a sound known as a “wheeze.” Typically, it is a “continuous sound” and occupies significantly more of the exhalation phase of breathing than the normal breath sounds do. This in large part accounts for the apparent “prolonged exhalation” often reported by clinicians when describing the signs observed in a child with flow limitation sufficient to cause a wheeze.

Those with long standing reduced lung function due to chronic disease often do not wheeze unless stressed by, for instance, exercise. A well-controlled asthmatic who suddenly develops airways narrowing might wheeze despite having an FEV1 greater than a patient of the same age with chronic poor control. It is not rare to see a young person report they are “fine” and note a “clear” chest only to find their FEV1 is 51% predicted with 45% bronchodilator responsiveness. This lack of wheeze in the face of clear airflow limitation may be related to changes in respiratory mechanics that are adopted to minimize wasted effort possibly incorporating braking of the chest wall recoil. As Meslier et al. noted “*wheezes are always accompanied by flow limitation but flow limitation is not necessarily accompanied by wheezes*.”

Fine **crackles** (crepitations) are due to respiratory units “snapping” open. The units do not open synchronously accounting for the discontinuous nature of the “crackles.” Classically this is a sign of “pneumonia” with the fluid filled alveoli and associated ducts opening up during inhalation. However, in the majority of infants under 6 months of age a viral infection of the conducting airways (bronchitis extending to the bronchioles i.e., bronchiolitis') crackles are generated, presumably due to snapping open of airways which close during exhalation due to intra luminal secretions (the snotty lung).

With the rapid physical growth of the airways in the 1st year of life airways close less often and the impact of the secretions becomes increasingly manifest as a wheeze due to flow limitation in the secretion filled airways.

A further common sound in infants and young children is the sound called a “*ruttle*” in parts of the U.K. (156, 158–160) and which was probably termed a rhonchus by many in the past though the same term has been used by as many to describe a wheeze heard with a stethoscope. “Ruttles” are harsh non-musical inspiratory and expiratory sounds heard with the naked ear are common in early childhood becoming very uncommon beyond 18 months of age. Generally they are more prominent on inspiration but occur in both phases They are often dismissed by clinicians as “transmitted sounds” (a fairly useless terms as all sounds are transmitted from somewhere) a term used in this context to signify them as of no importance suggesting they are being generated by secretions in the throat. This confusion probably arises from the *rattle* produced by patients accumulating secretions in their throat such as stroke victims and some with severe vertebra palsy. Alternatively, they are described as a “wheeze” as many clinicians only recognize a wheeze and crackles and decide they are not “crackles,” particular if audible without a stethoscope. However, these sounds are generated, as Lannec noted, by secretions in the large airways and commonly they cause vibrations palpable on the chest.

The auscultatory equivalent are *harsh inspiratory and expiratory* sound, usually louder on inspiration and which can be heard without a “ruttle” in older subjects.

A point often forgotten by clinicians is that there can be a significant amount of secretions in the airways with **no adventitial sounds** being generated. A good example would be the patient with CF or other form of a persistent bacterial bronchitis who has a chronic wet cough but is falsely reassured that “*it is just a virus*” because they apparently have a “clear” chest.

Given that both airflow limitation and significant secretions in the airways can be associated with a “clear chest” *the lack of added respiratory sounds is not necessarily a reassuring finding* even though many non-specialists appear to believe this.

An example of the correlation between the auditory signs and pathology would be a patient admitted with a moderately severe exacerbation of asthma. At presentation the patient will in all probability be wheezing, have a dry cough and demonstrate other signs of airways obstruction such as tachypnoea, hyperinflation and subcostal recession. Over a few days, having responded to corticosteroids the respiratory distress and wheeze will resolve but commonly the harsh inspiratory and expiratory sounds associated with excessive airways secretions together with a wet cough persist while the airways secretions that accumulated during the exacerbation are cleared. This is presumably the reason for persistent low saturations in some subject even when they feel “much better.”

## Viral LRTIs–The Same Pathology Generating Different Clinical Signs/Different Pathology Generating Very Similar Clinical Signs

**Figure 2:**
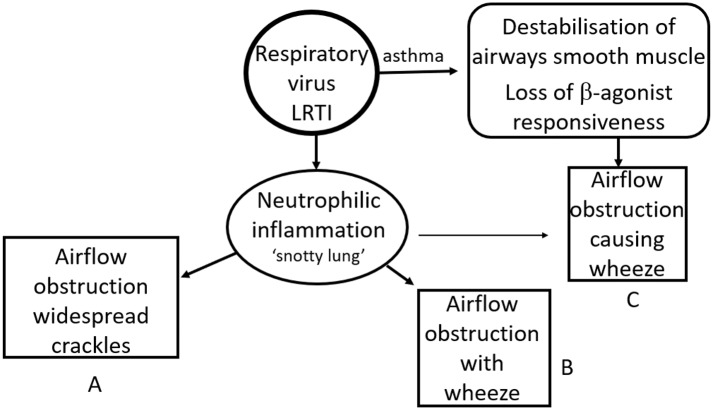
Impact of age and disease on clinical signs. An intense neutrophilic response to a respiratory virus can lead to significant airflow limitation. This typically causes widespread crackles predominantly in younger infants [GpA] and wheeze in older infants and preschool children [GpB]. The same clinical picture can be seen in an asthmatic preschool child with a viral exacerbation of asthma [GpC]. The incidence of children in GpA falls through the pre-school years while the prevalence of asthma increases from late infancy such that most children with a viral respiratory infection, airflow obstruction and wheeze at 15 months will be in GpB while the majority of those with the same clinical picture at 6 years of age will be in GpC.

In infants and pre-school children with a significant viral LRTI and evidence of airways obstruction adventitial breath sounds are common. The sound generated vary significantly with age such that a LRTI caused by a virus such as RSV or hRV inducing a similar neutrophilic response may generate widespread crackles in a 5-months-old infant and a clear expiratory wheeze in a 14 months old (153). The same pathology generates a different clinical picture in large part due to physical and physiological growth and development (Figure 2).

Conversely a 14 months old with asthma and a viral respiratory tract infection is able to generate a wheeze due to ASM constriction even if the neutrophilic response and consequent airways secretions are relatively mild and not sufficient to generate symptoms in their own right. Hence the same clinical picture is induced by two very different pathologies (see Figure 2).

The “wheeze” is a sign not a diagnosis (as in a “pre-school wheeze”) and is no more associated with a specific underlying pathology than is a limp. If all limps were simply labeled “childhood limp” then most children would not receive the treatment they require. The same applies to infants and young children with respiratory virus associated conducting airways obstruction.

## All That Wheezes is Not Wheeze

Numerous studies involving infants, children and adults have shown that even when clinicians are examining the patient at the same time agreement as to the presence or absence of a clinical sign is little better than chance in most cases (160–166). Hence when one clinician reports a child is wheezing a second clinician would be wise to wonder what the first clinician actually heard. Similarly, when parents report that their child had a “*wheeze*” (a term that does not exist in many languages) it is far from clear what they are describing with many using the term for any noisy breathing or indeed to describe apparent difficulty in breathing in the absence of any added sounds (167–179). Chevalier Jacksons aphorism needs to be updated to be accompanied by “*and all that wheezes is not wheeze*.”

The section on breath sounds highlights some of the challenges in using “breath sounds” to aid “diagnosis” in infants and young children with a viral LRTI. Flow limitation may cause a wheeze; excessive fluid in the airway may cause crackles in distal airways and a “ruttle” in more central airways; a mixture of signs is not rare and signs can change over short periods of time with or without the effects of a cough Conversely flow limitation and/or excessive airways secretions are frequently present occur despite an apparently “clear chest.”

Trying to base diagnostic and treatment decisions on the presence of certain added breath sounds is clearly fraught with challenges and should be viewed, at best, as unreliable.

## Implications for Diagnosis and Treatment of “Pre-school Wheeze”

The logical conclusion from the above information is that viral LRTIs vary significantly in severity and in the signs elicited despite a common underlying neutrophil dominated inflammatory response. The severity of symptoms can be enhanced by a variety of comorbidities including asthma. If a pre-school child with a an apparent viral LRTI wheezes there are a variety of conditions that can produce a similar picture including a severe bronchitis (which given the continuity of the conducting airways will involve the bronchioles), a viral exacerbation of asthma [in the absence of severe viral induced inflammation], a viral LRTI with a condition such as CLD of prematurity or significant tracheobronchomalacia. A pre-school child with an exacerbation of asthma or a “wheezy bronchitis” are both likely to have evidence of a respiratory viral infection in the form of coryza, a cough, increased work of breathing with a wheeze with poor response to salbutamol (characteristic of a viral exacerbation of asthma).

As noted above we have tended to go around in circles during the past four decades (37). Statements such that “*one cannot diagnose asthma under the age of 5 years (or under 2 years) of age*” and the use of terms such as “*reactive airways disease*” or “*viral wheeze*” *and* “*pre-school wheeze*” represent a lack of clarity in our approach to these common conditions. As noted above, these terms are the equivalent of labeling a child “*a limping child*” and failing to seek a more accurate diagnosis. As with the limping child a more accurate diagnosis aids appropriate therapy. An asthmatic will benefit from systemic steroids during an acute exacerbation while a child with a “*wheezy bronchitis*,” that is a child with a viral LRTI with an accompanying “wheeze” but without significant bronchoconstriction, will not. The former may also benefit significantly from regular ICSs if their symptoms are frequent or troublesome in contrast to the “wheezy bronchitic.” It should also be noted that some young pre-school children with PBB appear to develop airways obstruction and noisy breathing which may be a “ruttle” or indeed true wheeze during an inter-current viral LRTI resulting in an exacerbation.

However, coming to a firm diagnostic conclusion is far from straight forward and a clinician must be prepared to be humble enough to limit their conclusion to labels such as *possible asthma* or *probable wheezy bronchitis* based on a careful assessment of the history and examination of the individual child. As noted above, few appear to develop asthma in infancy. However, an increasing proportion of those with an apparent viral infection and airways obstruction (with or without wheeze) will be asthmatic with increasing age from 1 to 6 years of age. Age alone is not sufficient to be confidence of a diagnosis nor are the presence of other “markers” such as a family history of atopy or indeed family history of asthma. “Interval symptoms” are also difficult to interpret with a child attending childcare experiencing frequent infections of varying severity with little in the way of intervals while milder asthmatics often have few interval symptoms though they may take several weeks for symptoms to settle following a cold.

Reynolds and Cook recognized the challenge of identifying the minority of asthmatics amongst the entirety of pre-school wheezing children in their review of 1963 (171) noting “*As most pediatricians know, bronchiolitis is the commonest acute lower respiratory tract infection necessitating hospitalization in infants under the age of 1 year. Yet the lack of clear definition of the illness has been associated with marked confusion concerning its management*.” They went on to assert that “*Much of the confusion about management results from the fact that there are probably two groups of patients: (1) those with obstructive disease resulting entirely from infection, thickening of bronchiolar walls and intrabronchial secretion and (2) those with a predisposition to asthma who develop obstruction as a result of both inflammation and bronchospasm. These two groups cannot be readily distinguished …it would appear that most patients fall into the former group*.” Shields et al. produced striking confirmation of this dichotomy in their bronchoscopic studies more than 20 years ago (172, 173). The difficulty identified by Reynolds & Cook continues to challenge clinicians today. Accurate diagnosis will help ensure that those with asthma (in whom airways smooth muscle homeostasis is impaired (135)) receive appropriate therapy such as b-agonists and corticosteroids steroids while those with viral LRTIs (with or without “wheeze”) in whom there is no significant bronchoconstriction are not given unnecessary and potentially harmful medication. A number of those included in case reports of adrenal suppression causing hypoglycaemia and sometimes death in pre-school children resulting from the use of high dose inhaled steroids were subsequently shown not to have asthma.

## Design of Most RCTS Simply Compound the Confusion

Many interventional RCTS have been undertaken in this population of children. The majority have included those presenting acutely with respiratory distress and “wheeze” or who have “recurrent wheeze” (174–182). As such the data generated from all this effort is at best equivocal due to the failure to accurately characterize distinct patient groups. Such studies would be analogous to undertaking a trial of 4 weeks of intravenous antibiotics for “childhood limp.” While there are clearly some who would benefit in that their septic arthritis or osteoarthritis would be treated the effect is likely to be lost in the mass of musculoskeletal problems. Such a study might conclude there is no statistical benefit in using antibiotics to treat any child with a limp. A similar example concerns trials of the first effective inhaled steroid, beclomethasone which was apparently trialed by a Medical Research Council (MRC) who reported it did not work. The treatment was saved from the bin by Dr. Morrow-Brown who had carefully characterized his asthmatic patients on oral steroids. He demonstrated that the inhaled beclomethasone could provide excellent control with far less risk of side effects than the oral steroids (183). The MRC presumably including a mish mash of respiratory diagnoses losing the signal from the asthmatics in the noise of the patients with COPD and the like.

As noted previously there has been a tendency to go around in circles when it comes to the use of medications to treat pre-school children with an apparent viral respiratory tract infection and “wheeze.” In the late 1980's there was a big push to “stop denying pre-school children asthma medication” because children with “wheezy bronchitis” were in reality a form of asthma (based on information from school age children). Two decades later the prevailing view was that steroids had no place in the management of “pre-school wheeze.” Most studies have found no significant benefit while others have found a marginal benefit. Given the considerations outlines above this is perhaps unsurprising given that these studies take all comers and do not attempt to characterize the nature of a child's illness. If the majority, say 85% of those included, have a viral “wheezy bronchitis” and only 15% have asthma any signal in the asthmatics will be largely obscured because of the lack of any efficacy in the majority of subjects. In a different cohort with perhaps 60% with asthma discernible effects may be observed even if a large minority do not respond. It is likely that a 3 years old with asthma will respond to the same treatment as a 6 years old–the challenge is to pick the 3 years old asthmatic from the larger group with recurrent symptoms not attributable to asthma.

## Proposed Terminology

**Figure 3:**
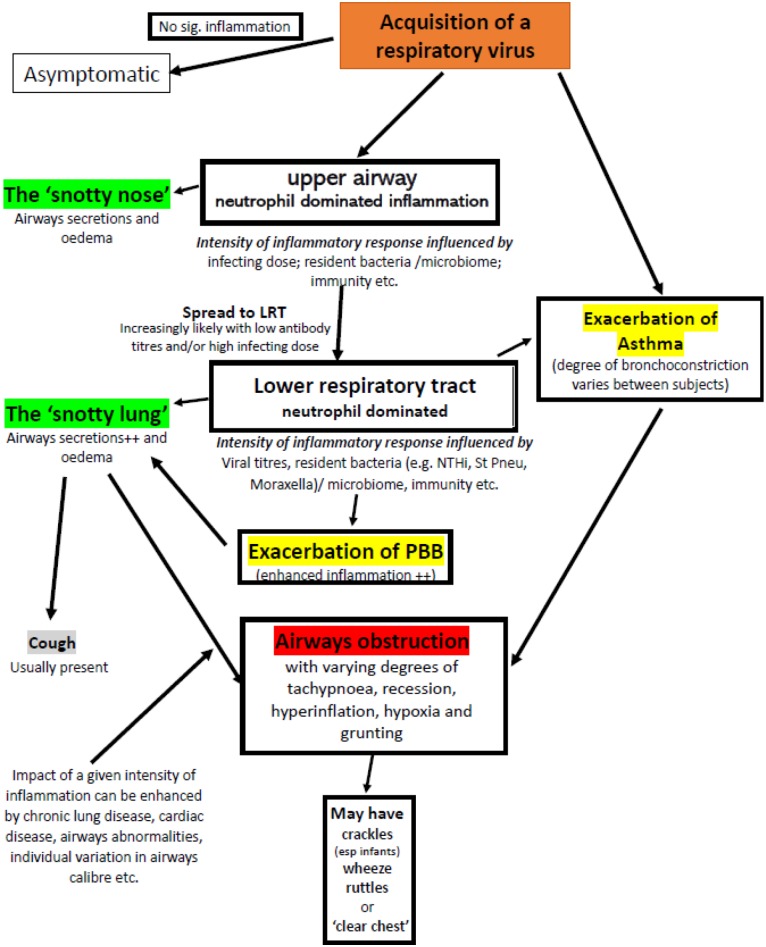
Factors that may contribute to airflow obstruction in an infant or pre-school child following acquisition of a respiratory virus. Adventitial respiratory sounds may or may not be present. Exacerbations of asthma and PBB require specific therapy, the majority of episodes require good supportive care appropriate to the severity of symptoms.

Given that the *same pathology can generate apparently very different clinical signs* (such as crackles, wheeze, and ruttle) and *very different pathologies can produce very similar clinical signs* (wheezy bronchitis and asthma) (Figure 3) it is perhaps time to revise our terminology to improve clarity of thought when faced with a young child with an apparent viral infection and significant airways obstruction.

Terms such as RAD, bronchiolitis, wheeze associated viral infection, viral wheeze, pre-school wheeze etc. should be abandoned. While wheezy bronchitis would appear to clearly signify a viral bronchitis with associating wheezing in the absence of significant bronchoconstriction it has been used in the past to describe asthma and hence perhaps should not be resurrected. Moreover, significant flow limitation can be present without wheeze despite the same underlying pathology and hence wheeze is not the defining feature of viral induced neutrophilic inflammation leading to airflow obstruction. The following is likely to be far more helpful in clarifying diagnostic and therapeutic.

When faced by a pre-school patient with an apparent respiratory viral infection accompanied by signs suggestive of obstruction within the conducting airways (e.g., tachypnoea, recession, hyperinflation, use of accessory muscles) the diagnosis will be one of

1. an acute viral induced LRTI in a previously well-infant/child with no apparent co-morbidities
2. an acute viral induced LRTI in a child with pre-existing comorbidity such as (a) chronic lung disease of prematurity or other form of chronic lung disease (b) cardiac disease (esp. left to right shunts) (c) an airways abnormality such as tracheobronchomalacia
3. an exacerbation asthma or PBB.

Those in groups i) and ii) are unlikely to benefit from any specific therapy (though some may use antivirals for at risk infants with influenza) and benefit from supportive therapy as noted by Reynolds & Cook more than 50 years ago (171) ***(viral bronchitis*** **+*/*−** ***a structural comorbidity)*.**

The importance of considering co-morbidities (group ii) is that they may influence both short to medium prognosis and the approach to supportive care. Many with a significant co-morbidity are likely to present multiple times with significant symptoms due to the high rates of respiratory viral infections in early life and the effect of the co-morbidity on the severity of symptoms. I contrast, while some otherwise apparently well-infants appear to have an unexplained tendency to experience recurrent significant symptomatic LRTIs they are much smaller proportion of the total group (though numerically may be greater). In terms of supportive care a young child with significant tracheobronchomalacia and recurrent admissions may benefit from early use of positive pressure support such as the use of high flow nasal canulae while a child with a lobar emphysema's condition may deteriorate with injudicious use of positive pressure support.

Those in group iii) **(exacerbation of asthma or PBB)** will benefit from specific therapy. However, as noted above, it is very difficult without taking a careful history to be confident of a likely diagnosis in a child without a previous definitive diagnosis. Therefore, the use of qualifiers such as possible or probable may need to be used as in “*a probable exacerbation of asthma*.” Of course, conditions such as PBB and asthma or PBB and TBM can co-exist.

In this scheme “*wheeze*” is simply one of a number of adventitial sounds that can accompany airways obstruction induced by a virus and in itself carries little diagnostic weight. Of those presenting at 15 months of age most will be in group i) whilst amongst 5-years-old patients with a similar clinical picture group iii) patients will predominate.

## How to Identify Those Children WHO Benefit From Specific Therapy?

Doctors are generally most comfortable, and effective, when they can act on the results of tests. The great challenge for pediatric pulmonologists and others looking after young children with acute and/or chronic respiratory illnesses is that for the majority of conditions we do not have a simple or indeed complex tests. There are a few exceptions such as neonatal screening, sweat tests and genetics for cystic fibrosis and cilia function and genetics for primary ciliary dyskinesia but for the common conditions we are still reliant on a careful clinical history and where appropriate a response to treatment. Of all the medical specialties effective care in pediatric respiratory medicine is perhaps the most reliant on effective history taking and extremely careful assessment of the response to a trial of treatment which in many cases is the most accurate diagnostic test.

Discussing the features in the history (examination is rarely helpful) that might help distinguish asthma, PBB, frequent recurrent viral infections and other conditions is beyond the scope of this review but has been discussed elsewhere (87, 88, 135). However, a history can only help with a provisional diagnosis–possible or probable asthma or PBB for example but a definite diagnosis should only be made when a dramatic and unequivocal response to therapy is observed at a time point when such a response would be anticipated (e.g., 2 weeks of high dose antibiotics for PBB, 5 days of oral steroids or 5–8 weeks for inhaled corticosteroids for asthma). The response from the parent should be unequivocal “*I have my boy back*,” “*she is a new girl*” not “*he/she is coughing less and sleeping better*” which may simply be the typical regression to the mean after an exacerbation.

Sadly, until effective diagnostics are available, and there is no immediate prospects of this for the common conditions affecting pre-school children, those looking after young children with recurrent and persistent respiratory illness need to be comfortable living with uncertainty and, more importantly, make the effort to keep reviewing ones provisional diagnosis until one can reach an accurate and definitive diagnosis. “*It is just another virus*,” “*try this inhaler, it is probably asthma*” are not acceptable.

Until we can develop a simple test for the diagnosis of asthma and other conditions such as PBB the significant on-going over and under diagnosis of asthma will persist particularly in pre-school children (135). We can in part reduce the inaccuracies in diagnosis by clarifying our terminology and thinking.

## Author's Note

Apologies to those working in non-English speaking countries that have been confused by the failure of English speaking physicians to standardize nomenclature across the spectrum of early respiratory tract infections. This article may clarify some of the causes for this confusion.

## Author Contributions

ME conceived and wrote the manuscript together with KD.

## Conflict of Interest

The authors declare that the research was conducted in the absence of any commercial or financial relationships that could be construed as a potential conflict of interest.
